# Metformin attenuates high-fat diet induced metabolic syndrome related osteoarthritis through inhibition of prostaglandins

**DOI:** 10.3389/fcell.2023.1184524

**Published:** 2023-05-02

**Authors:** Xiaonan Liu, Qiaoyue Guo, Lei Wang, Yiru Gu, Senxiong Meng, Yuan Gu, Bin Yu

**Affiliations:** ^1^ Division of Orthopaedics and Traumatology, Department of Orthopedics, Nanfang Hospital, Southern Medical University, Guangzhou, China; ^2^ Department of Endocrinology, Endocrinology Research Center, Xiangya Hospital of Central South University, Changsha, China; ^3^ Bloomberg School of Public Health, The Johns Hopkins University, Baltimore, MD, United States

**Keywords:** high-fat diet, prostaglandins, macrophage, metformin, osteoarthritis, subchondral bone

## Abstract

High-fat diet induces bone marrow inflammation and osteoarthritis phenotype in knee joint, but the underlying mechanisms is unknown. Here, we report that high-fat diet induces aberrant bone formation and cartilage degeneration in knee joint. Mechanistically, a high-fat diet increases the number of macrophages and the secretion of prostaglandins in subchondral bone, promoting bone formation. Metformin treatment is able to decrease the number of macrophages and also the level of prostaglandins induced by high-fat diet in subchondral bone. Importantly, metformin rescues aberrant bone formation and cartilage lesions by decreasing the number of osteoprogenitors and type-H vessels, which also results in relief of osteoarthritis pain response. Thus, we demonstrate prostaglandins secreted by macrophages may be a key reason for high-fat diet induced aberrant bone formation and metformin is a promising therapy for high-fat diet induced osteoarthritis.

## Introduction

Osteoarthritis is a common joint disease and one of the leading causes of pain response and disability in general population (Conaghan et al., 2019; Hunter and Bierma-Zeinstra, 2019). However, little is known about prevention and intervention of early-stage osteoarthritis due to complex etiology and limited understanding of potential mechanisms (Martel-Pelletier et al., 2016). As a result, there is no disease-modifying drug for this disease (Block, 2014; Conaghan et al., 2019). It has been noted that trauma played an important role in the development of cartilage damage. However, previous reports showed only about 12% diagnosed osteoarthritis cases belonged to post-traumatic osteoarthritis, suggesting involvement of other factors in the development of osteoarthritis (Su et al., 2022).

Recently, metabolic diseases were found to be vital contributors in osteoarthritis development (Collins et al., 2018; Misra et al., 2019; Mohajer et al., 2021). Metabolic syndrome is a cluster of conditions including high blood pressure, high blood sugar, excess body fat around the waist, and abnormal cholesterol levels (Furuta et al., 2023; Kassi et al., 2011). Studies have found that people with metabolic syndrome were more likely to have knee osteoarthritis than those without the metabolic condition (Liu et al., 2020; Puenpatom and Victor, 2009). Interestingly, although metabolic syndrome can cause obesity which will directly accelerate the “wear and tear” process of articular cartilage, patients with metabolic syndrome also have a higher risk of developing osteoarthritis in non-weight-bearing joints such as hands, suggesting that metabolic syndrome related osteoarthritis is not only caused by increased body weight but also changed bone metabolism microenvironment (Mohajer et al., 2021; Sanchez-Santos et al., 2019). Some studies have reported the link between metabolic syndrome and osteoarthritis could be due to chronic inflammation and metabolic dysfunction of bone marrow cells (Batushansky et al., 2022; Courties et al., 2017). Specifically, a high-fat diet was shown to increase the number of osteoclasts and shift the fate of BMSCs from osteoblast toward adipocyte lineage, contributing to increased bone marrow adiposity (Montalvany-Antonucci et al., 2018). Studies also found high fat det could increase the expression of plasma inflammatory factors, as well as infiltration of aggravative inflammatory cells (e.g., CD11^+^ T macrophages) in adipose tissue, promoting an inflammatory bone marrow microenvironment (Qiao et al., 2021).

Metformin is a widely recognized medication for type 2 diabetes that has been shown to have various other health benefits beyond glycemic control (Kulkarni et al., 2020; Mohammed et al., 2021). Recently, there has been growing interest in the potential of metformin in the management of osteoarthritis (Li D. et al., 2022; Li et al., 2020). Inflammation plays a significant role in the pathogenesis of osteoarthritis. Studies have reported that metformin may reduce the production of pro-inflammatory cytokines, such as interleukin-1β and tumor necrosis factor-α, which are known to play a crucial role in the development of osteoarthritis (Li D. et al., 2022; Li et al., 2020; Terkeltaub et al., 2011). Furthermore, metformin has been shown to inhibit the activation of nuclear factor kappa B, a transcription factor that regulates the expression of various pro-inflammatory genes (Hattori et al., 2006; Isoda et al., 2006). Recent studies have suggested that metformin may also have chondroprotective effects that could promote cartilage regeneration (Li D. et al., 2022; Li et al., 2020). *In vitro* studies have reported that metformin promotes the proliferation and survival of chondrocytes, the cells responsible for producing and maintaining cartilage (Wang C. et al., 2019; Xing et al., 2022). Moreover, metformin has been shown to downregulate the expression of ADAMTS5 and MMP1, two genes that are responsible for the degradation of cartilage (Schadler et al., 2021). Pain is the most common symptom associated with osteoarthritis, and it can have a significant impact on the quality of life of affected individuals (Conaghan et al., 2019). Of note, metformin has been shown to have analgesic effects that could potentially benefit patients with osteoarthritis (Li Z. et al., 2022; Na et al., 2021). Animal studies have reported that metformin reduces pain sensitivity in response to noxious stimuli (Li et al., 2020). Moreover, metformin has been shown to activate the adenosine monophosphate-activated protein kinase (AMPK) pathway, which is involved in pain modulation (Li et al., 2020). Despite the progress in the use of metformin in traumatic OA, few studies explored the use of metformin in metabolic syndrome related osteoarthritis. Whether metformin also reduces inflammation and pain response in metabolic osteoarthritis is unknown.

In this study, we characterized the joint phenotype of high-fat diet induced metabolic syndrome induced osteoarthritis and found that metformin was able to inhibit high-fat diet stimulated prostaglandins production and reverse aberrant bone formation in subchondral bone. In conclusion, our data showed metformin could emerge as a potential therapeutic option for the management of metabolic syndrome related osteoarthritis. Its anti-inflammatory effects, chondroprotective properties, and analgesic effects suggest that it could be a useful adjunct to current treatments for osteoarthritis. However, further studies are needed to fully understand the mechanisms of action of metformin in osteoarthritis and to determine its efficacy and safety in clinical trials.

## Results

### Evaluation of cartilage and subchondral bone phenotype in HFD-treated mice

**FIGURE 1:**
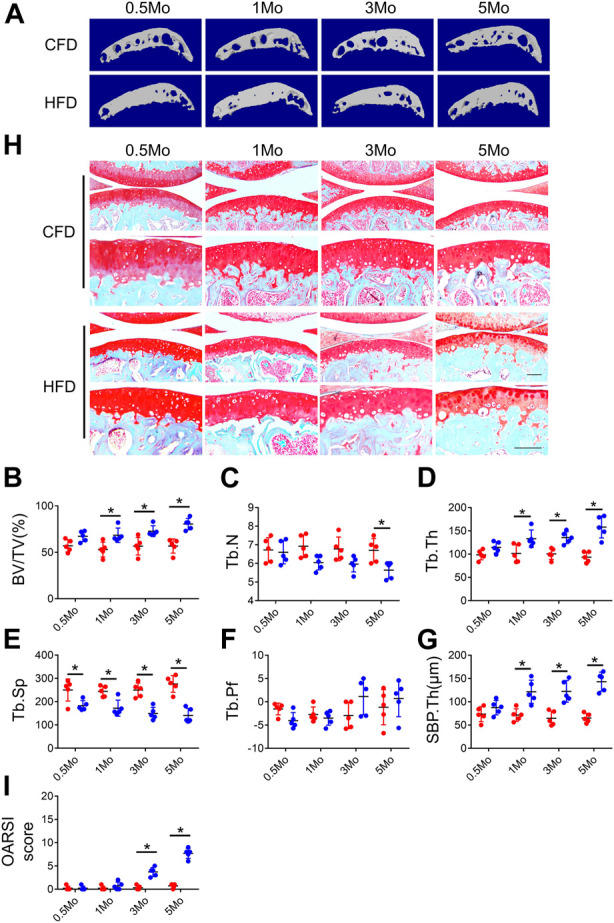
Evaluation of cartilage and subchondral bone phenotype in HFD-treated mice. Three-month-old C57BL/6 mice were fed a chow-food diet (CFD) or a high-fat diet (HFD) for 0.5, 1, 3, or 5 months. n = 6 mice per group. **(A–G)** Representative image of three-dimensional micro-computed tomography (μCT) images **(A)** and quantitative analysis of structural parameters of knee joint subchondral bone: bone volume/tissue volume (BV/TV, %) **(B)**, trabecular number (Tb.N, mm^−1^) **(C)** trabecular thickness (Tb.Th, mm) **(D)**, trabecular bone separation (Tb. Sp, mm) **(E)**, trabecular pattern factor (Tb. Pf, mm^−1^) **(F)**, and subchondral bone plate thickness (SBP. Th, mm) **(G)**. **(H, I)** Safranin O-fast green staining of the tibia subchondral bone medial compartment (sagittal view) **(H)** and calculation of Osteoarthritis Research Society International (OARSI) scores **(I)**. All data are shown as means ± standard deviations. *p < 0.05. Statistical significance was determined by unpaired, two-tailed Student’s t-test.

Previous studies have reported conflict results regarding knee joint osteoarthritis development in high-fat diet (HFD) condition (Brunner et al., 2012; Sansone et al., 2019). To evaluate whether HFD can induce OA phenotype, we first investigated the subchondral bone and cartilage phenotype at different timepoints during HFD treatment. Micro-CT analysis showed a dramatic increase in subchondral bone mass starting from 0.5Mo in HFD-treated group as evidenced by increased bone volume (BV)/tissue volume (TV) ratio, trabecular bone thickness (Tb.Th) and bone plate thickness (SBP.Th) in the medial part of the subchondral bone region, suggesting HFD induced rapid bone formation in the subchondral bone area (Figures 1A–G). A continued decrease in subchondral bone trabecular number was also observed, possibly because of disappearance of bone marrow cavities (Figure 1C). SOFG staining showed a mild cartilage degeneration starting at 3Mo and apparent thinning of the cartilage layer at 5Mo, resulting in higher scores at the above timepoints in OARSI scoring (Figures 1H, I). These results demonstrated HFD could induce a rapid subchondral bone change but rather mild cartilage degeneration in knee joint, suggesting that HFD could serve as a metabolic OA model that has different phenotypes compared to post-traumatic OA (PTOA) or rheumatoid arthritis (RA).

### Metformin reduces PGs level and cellular senescence in subchondral bone in mice treated with HFD

**FIGURE 2:**
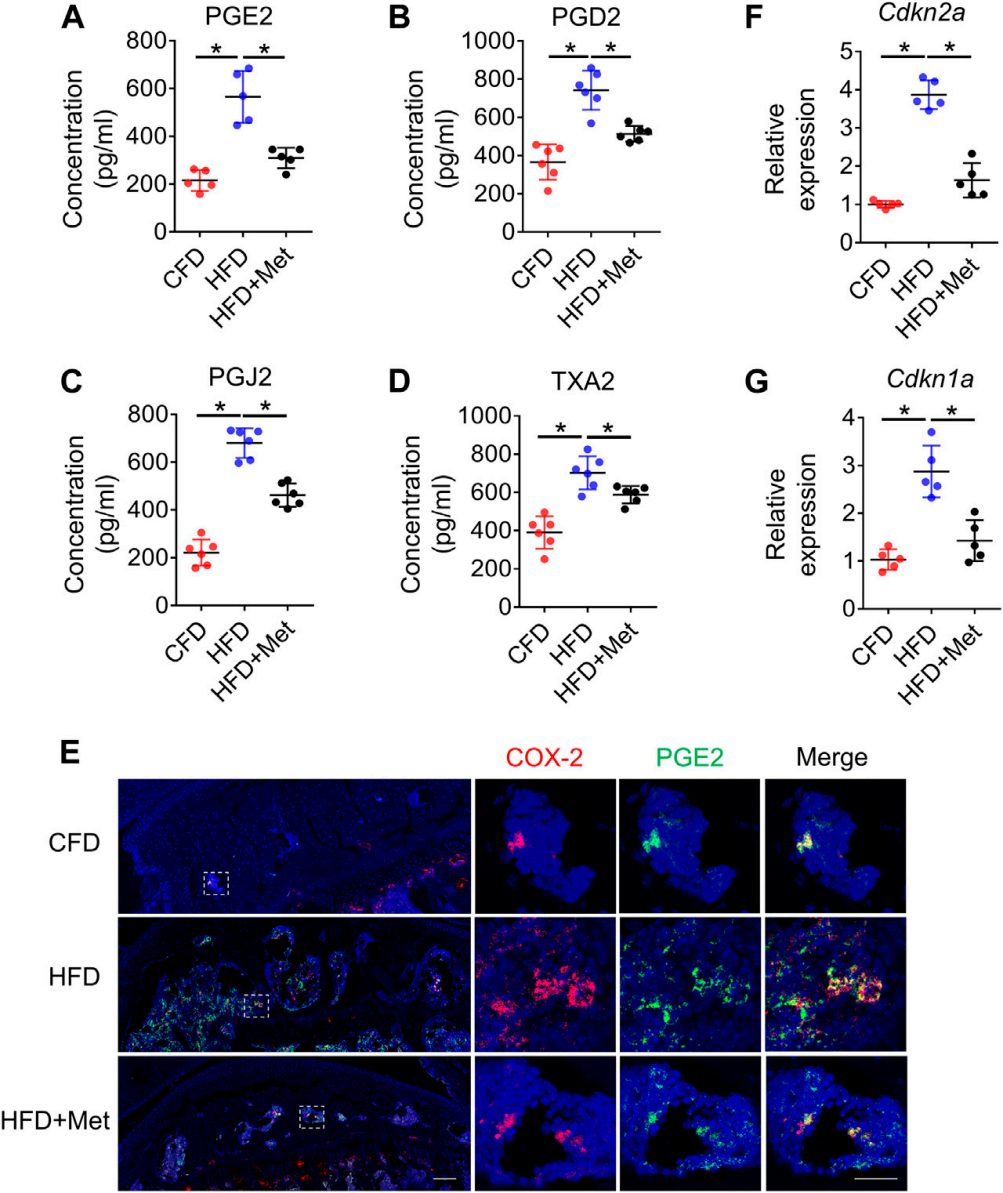
Metformin reduces PGs level and cellular senescence in subchondral bone in mice treated with HFD. Three-month-old C57BL/6 mice were fed a standard chow-food diet (CFD), HFD, or HFD with metformin (HFD + Met) for 3 months. n = 6 mice per group. **(A–D)** Relative protein concentrations of *PGE2* level **(A)**, *PGD2* level **(B)**, *PGJ2* level **(C)**, and *TXA2* level **(D)** in subchondral bone of mice treated with CFD, HFD, or HFD + Met. **(E)** Immunofluorescence staining of knee joint tissue sections with antibody against COX-2 (red) and PGE2 (green). **(F, G)** RNA was isolated from harvested tibial plateau and mRNA levels of *Cdkn2a* and *Cdkn1a* were measured by qRT-PCR analysis. All data are shown as means ± standard deviations. *p < 0.05. Statistical significance was determined by one-way ANOVA.

Several studies showed that prostaglandin production was the key reason for OA development and OA pain. To elucidate whether prostaglandins were also elevated in HFD induced metabolic OA, we examined the expression of prostaglandins in subchondral bone of HFD-treated mice. We selected 3-month as timepoint based on our previous finding that OARSI scores were evident starting from 3-month. Notably, we found a significant increase of 4 primary prostaglandins: PGE2, PGD2, PGJ2 and TXA2 in subchondral bone extract of HFD-treated mice (Figures 2A–D). Immunostaining further confirmed a colocalization and significant increase in COX-2 and PGE2 in the subchondral bone in HFD-treated mice (Figure 2E). As a previous study indicated HFD-induced senescent cells could also be an important source of PGE2 production, we also examined whether metformin was able to reduce senescence burden in tibia joint tissue (Su et al., 2022). Intriguingly, we found although HFD could significantly increase *p16* and *p21* expression in harvested tibial plateau, metformin could reduce the expression to almost base level, suggesting that metformin could also inhibit COX2-PGE2 production though inhibition of senescent cells (Figures 2F,G).

### Metformin reduces the number of COX-2^+^ macrophages in subchondral bone

**FIGURE 3:**
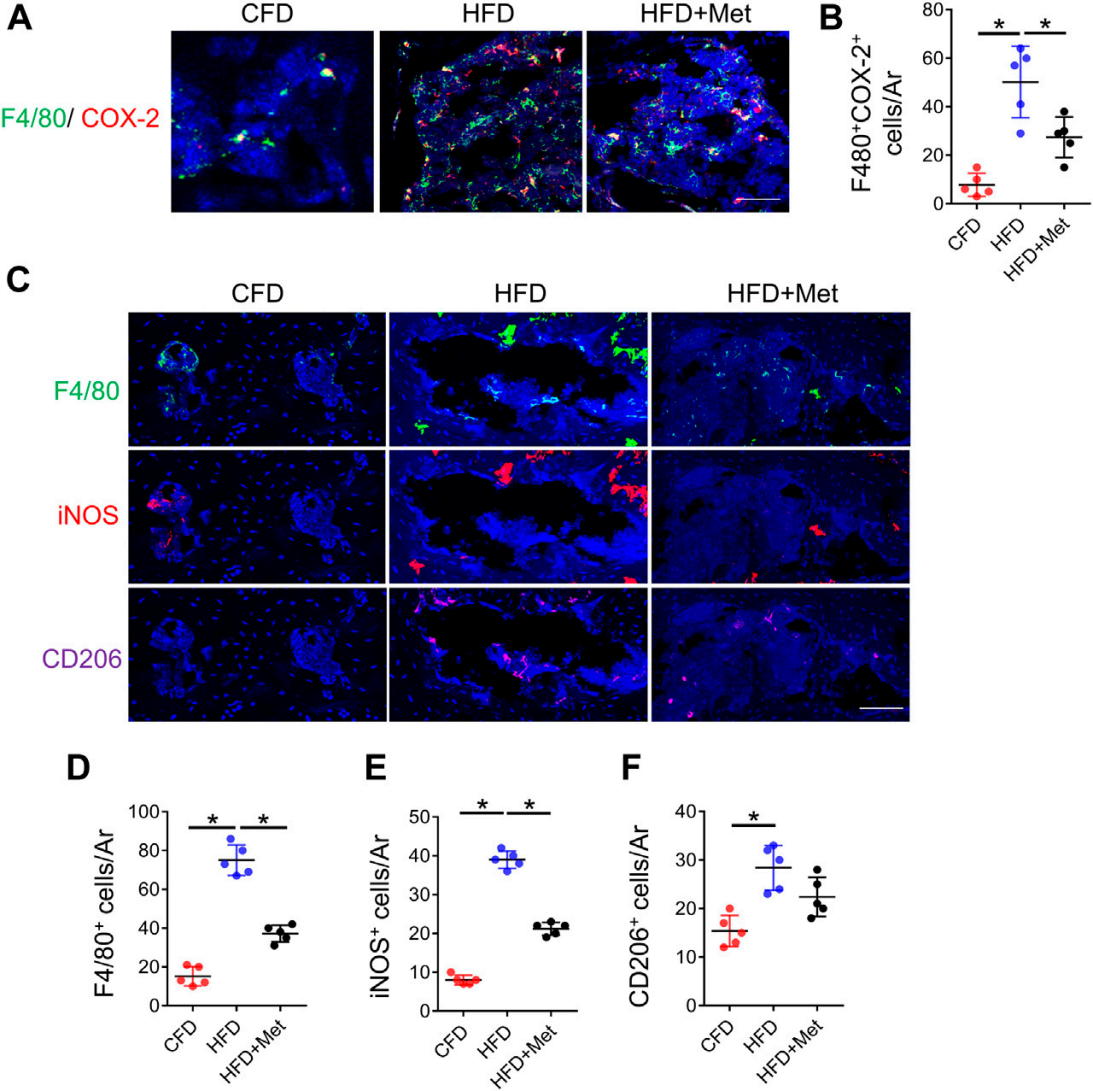
Metformin reduces the number of COX-2^+^ macrophages in subchondral bone. Three-month-old C57BL/6 mice were fed a standard chow-food diet (CFD), HFD, or HFD with metformin (HFD + Met) for 3 months. n = 6 mice per group. **(A, B)** Immunofluorescence double staining of COX-2 (red) and F4/80 (green) in **(A)** and quantification of the number of F4/80^+^COX-2^+^ cells in subchondral bone sections in **(B)**. **(C–F)** Immunofluorescence staining of F4/80(green), iNOS(red), and CD206 (purple) in **(C)**, quantification of the number of F4/80^+^ cells in subchondral bone sections in **(D)**, quantification of the number of iNOS^+^ cells in subchondral bone sections in **(E)**, and quantification of the number of CD206^+^ cells in subchondral bone sections in **(F)**. All data are shown as means ± standard deviations. *p < 0.05. Statistical significance was determined by one-way ANOVA.

Since HFD model can induce metabolic syndromes which can be treated with drugs targeting metabolism, for example, metformin, we tested if metformin was also able to reverse prostaglandin production in HFD-induced metabolic OA. Co-treatment of metformin with HFD for 3 months significantly reduced the level of PGE2, PGD2, PGJ2 and TXA2 in subchondral bone region (Figures 2A–D), which was also validated by immunostaining of COX-2 and PGE2(Figure 2E). Previous reports have showed that monocyte-macrophage lineage cells were one of the main sources of prostaglandins in bone marrow (Hayes et al., 1992). To explore the potential source of elevated prostaglandins in HFD-treated subchondral bone, we conducted co-staining of F4/80 and COX-2. The result demonstrated 70.80% ± 10.47% COX-2-positive cells were F4/80-positive cells, suggesting elevated prostaglandins were mainly produced by macrophages (Figures 3A, B). However, the number of F4/80 and COX-2 double-positive cells decreased dramatically upon metformin treatment, suggesting metformin was able to inhibit prostaglandin production in macrophages. Immunostaining of macrophage markers showed that metformin could reduce the toal number of macrophages as showed by decreased F4/80-positive cells (Figures 3C, D). Moreover, metformin was able to inhibit the specific M1-type macrophages (iNOS+) but not M2-type macrophages (CD206+), indicating metformin prevented generation of the pro-inflammatory environment in subchondral bone in HFD-treated mice (Figures 3C, E, F).

### Metformin improves HFD-induced metabolic syndrome related osteoarthritis

**FIGURE 4:**
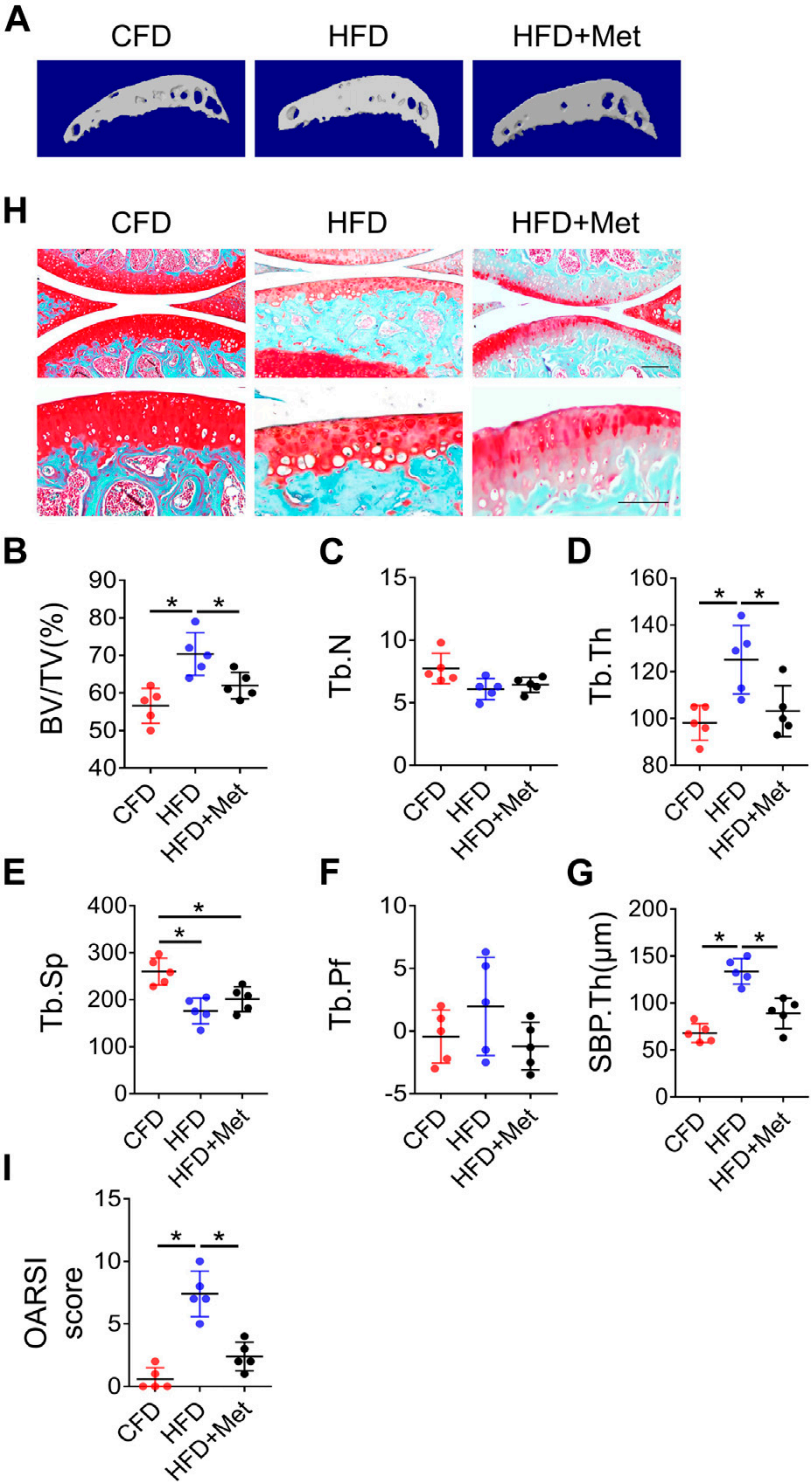
Blocking PGs improves HFD-induced metabolic OA. Three-month-old C57BL/6 mice were fed a standard chow-food diet (CFD), HFD, or HFD with metformin (HFD + Met) for 3 months. n = 6 mice per group. **(A–H)** Representative image of three-dimensional micro-computed tomography (μCT) images **(A)** and quantitative analysis of structural parameters of knee joint subchondral bone: bone volume/tissue volume (BV/TV, %) **(B)**, trabecular number (Tb.N, mm^–1^) **(C)** trabecular thickness (Tb.Th, mm) **(D)**, trabecular bone separation (Tb. Sp, mm) **(E)**, trabecular pattern factor (Tb. Pf, mm−^1^) **(F)**, and subchondral bone plate thickness (SBP. Th, mm) **(G)**. **(H, I)** Safranin O-fast green staining of the tibia subchondral bone medial compartment (sagittal view) **(H)** and calculation of Osteoarthritis Research Society International (OARSI) scores **(I)**. All data are shown as means ± standard deviations. *p < 0.05. Statistical significance was determined by onE-way ANOVA.

Based on above findings, we further investigated whether metformin was able to reverse HFD-induced OA phenotype. In accordance with our previous results, 3-month HFD treatment significantly increased bone volume in subchondral bone. However, this phenotype was alleviated by metformin treatment as evidenced by reduced BV/TV, Tb.Th and SBP.Th (Figures 4A–G), which is consistent with OARSI score (Figures 4H,I). Of note, metformin could only partially rescue the effect of HFD on subchondral bone, suggesting that other mechanisms also function in the HFD-induced OA development.

### Metformin rescues aberrant vessel and bone formation in HFD-treated mice

**FIGURE 5:**
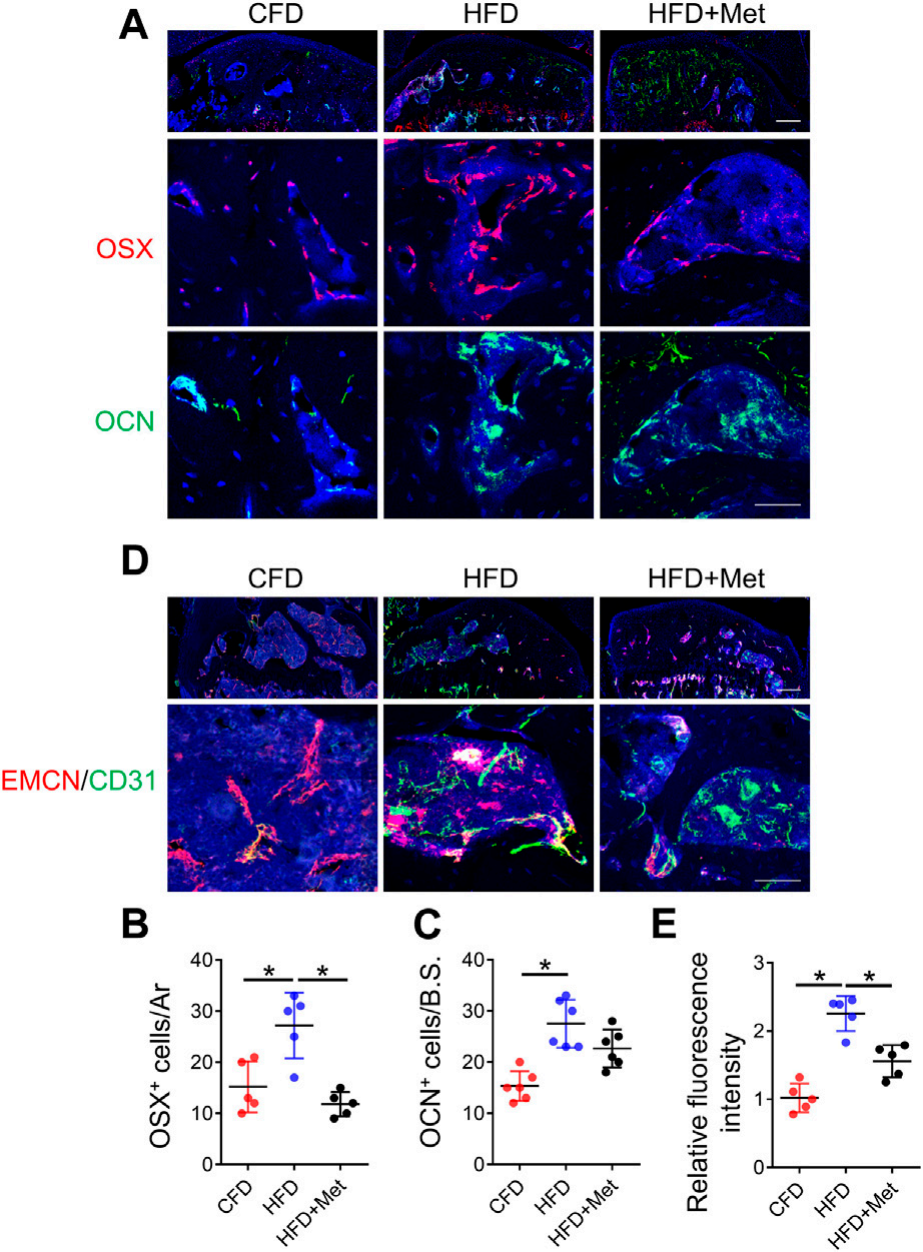
Metformin rescues aberrant vessel and bone formation in HFD-treated mice. Three-month-old C57BL/6 mice were fed a standard chow-food diet (CFD), HFD, or HFD with metformin (HFD + Met) for 3 months. n = 6 mice per group. **(A–C)** Immunofluorescence staining of knee joint tissue sections with antibody against Osterix (OSX, red) and osteocalcin (OCN, green) **(A)**, quantification of the number of OCN^+^ cells in subchondral bone sections in **(B)**, and quantification of the number of OCN^+^ cells in subchondral bone sections in **(C)**. **(D–E)** Immunofluorescence double staining of knee joint tissue sections with antibody against endomucin (EMCN, red) and CD31 (green) **(A)** and quantification of the fluorescence intensity of EMCN^+^ CD31^+^area in subchondral bone sections in **(E)**. All data are shown as means ± standard deviations. *p < 0.05. Statistical significance was determined by one-way ANOVA.

Previous studies showed angiogenesis played a vital role in OA development (Su et al., 2020). We examined whether metformin reduced aberrant bone formation by decreasing osteoblasts and blood vessels. Immunofluorescence staining showed while HFD treatment caused significant increase in both Osterix (OSX)^+^ osteoblast progenitors and Osteocalcin (OCN)^+^ mature osteoblasts, metformin co-treatment reduced the number of OSX^+^ osteo-progenitors to base level comparable to CFD group (Figures 5A, B). The number of OCN^+^ mature osteoblasts was also reduced by metformin co-treatment (Figure 5C). We also detected the number of type H vessels, the bone-formation related blood vessel, in subchondral bone in HFD treated mice. 3-month HFD treatment increased EMCN^+^CD31^+^ type H vessels in subchondral bone, whereas metformin markedly reduced this angiogenesis process (Figures 5D, E).

### Metformin rescues HFD induced OA pain

**FIGURE 6:**
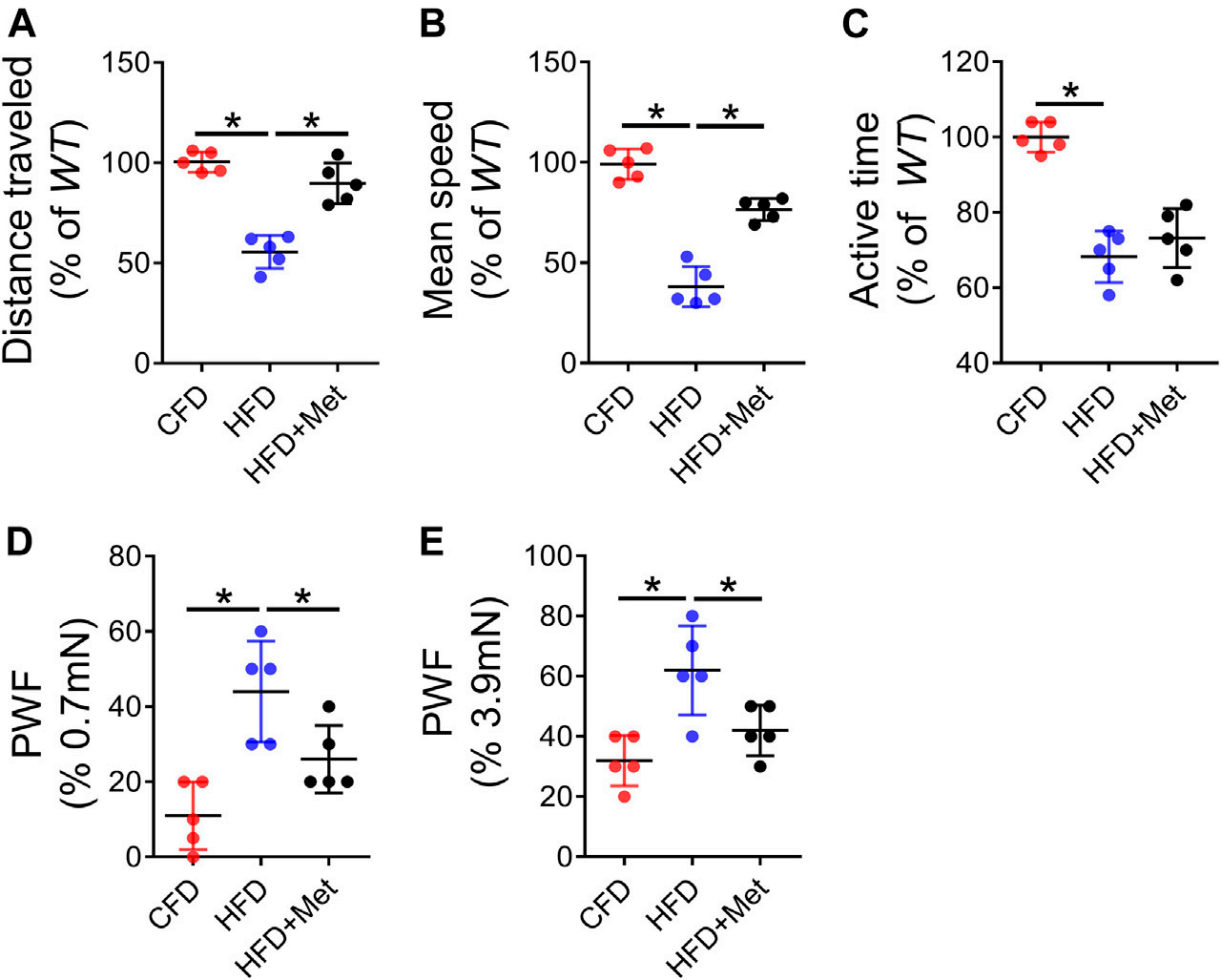
Metformin rescues HFD induced OA pain. **(A–C)** Parameters of voluntary wheel running for mice which were fed a standard chow-food diet (CFD), HFD, or HFD with metformin (HFD + Met): distance traveled **(A)**, mean speed **(B)**, and active time **(C)**; n = 5 mice per group. **(D–E)** Parameters of von fray test for mice which were fed a standard chow-food diet (CFD), HFD, or HFD with metformin (HFD + Met): paw withdraw frequency (PWF) at 0.7 mN stimulation **(D)** and PWF at 3.9 mN stimulation **(E)**. All data are shown as means ± standard deviations. *p < 0.05. Statistical significance was determined by one-way ANOVA.

We finally investigated pain response using multiple behavior tests. We first monitored basic spontaneous physical activity including distance traveled, maximum speed of movement and active time per 24 h to reflect potential overall effect of joint pain on mice. We found 3-month HFD treatment significantly reduced these parameters as compared to CFD treated mice (Figures 6A–C). The mechanical hyperalgesia of the hind paw is also increased in HFD-treated mice relative to CFD-treated mice as measured by von Frey analysis, suggesting increased pain response induced by HFD-induced OA. Of note, metformin co-treatment in mice showed significant improvement in spontaneous activity and mechanical hyperalgesia (Figures 6D, E). These results suggest metformin was able to attenuate HFD induced OA phenotype and related pain response.

## Discussion

Osteoarthritis remains one of the leading causes of disability and joint pain among middle-aged and old people (Sun et al., 2021). It was estimated that approximately 10% of men and 18% of women had symptomatic OA, which significantly decreased their quality of life (Allen et al., 2022). However, due to the complex causes of disease onset, there is no disease-modifying drug for this disease (Block, 2014; Conaghan et al., 2019). Current animal models of osteoarthritis mainly focused on pathological changes in traumatic conditions such as ACLT or DMM. Whereas the phenotype changes in subchondral bone and cartilage in nontraumatic osteoarthritis were poorly documented. In our study, we found that high-fat diet treatment could induce rapid bone formation in subchondral bone. But the osteoarthritis phenotype, of note cartilage degeneration, only occurred after a long-term high-fat diet treatment (>3Mo). Moreover, the cartilage changes in high-fat diet treatment group also appears mild compared with in traumatic models, as we observed loss of collagen content and over-hypertrophy of some chondrocytes rather than severe erosion of cartilage surface (Jeon et al., 2017). These phenotypes of metabolic syndrome related osteoarthritis is distinct from the phenotypes of traumatic models (Cui et al., 2016; Zhen et al., 2013; Zhu et al., 2019). Specifically, traumatic model will induce early bone loss and later uneven bone formation in bone marrow of subchondral bone, which leads to subsequent cartilage erosion (Zhen et al., 2013). However, in high-fat diet model, aberrant bone formation was found distributed rather evenly in bone marrow of subchondral bone (Su et al., 2022). This may explain the mild nature of cartilage degeneration in this model since the mechanical property of the subchondral bone-cartilage unit is still intact to some extent. A thickened subchondral bone may not serve as a good cushion for the cartilage layer as in wild-type mice and cause cartilage degeneration gradually. However, given that high-fat diet also has systematic effect like glucose level, fatty acids metabolism and inflammation, which could affect synovial fluid and thus chondrocyte metabolism, it still remains unclear whether the long-term cartilage damage is due to subchondral bone changes or direct effect on chondrocytes (Gross et al., 2014; Xie and Chen, 2019).

The COX2-PGE2 axis has been proved to be a vital pathway in bone homeostasis (Chen et al., 2019; Raisz, 1999). In long bone, COX2-PGE2 expression stimulates osteogenesis process through MAPK pathway or Wnt pathway and thus promotes fracture healing (Liedert et al., 2010; Minamizaki et al., 2009). Interestingly, *in vitro* study confirmed that mechanical load could increase COX2 expression in bone marrow and promoted bone formation, suggesting COX-2 level was regulated by both metabolic and mechanical factors (Liedert et al., 2010). In recent studies, the COX2-PGE2 axis was proved to play a central role in bone interoception which mediated sensing of bone volume by CNS system (Lv et al., 2022). It is well recognized that the subchondral bone is an important part of the weight bearing unit of joint. Various studies have demonstrated COX2-PGE2 axis could modulate the structure of subchondral bone during the onset of different types of OA (Su et al., 2022; Tu et al., 2019). In our study, we found that COX-2-PGE2 axis was also activated in metabolic syndrome related osteoarthritis, indicating high-fat diet elicited a inflammatory local microenvironment in subchondral bone, which may explain the aberrant bone formation at this site. As metformin was proved to diminish inflammation in metabolic diseases, we also tested if metformin was able to decrease COX-2-PGE2 expression in subchondral bone. We found metformin could reduce the production of major forms of prostaglandins such as PGE2, PGJ2, PGD2 and TXA2. Of note, as we found increased macrophages in high-fat diet treatments, the number of macrophages, especially M1 type macrophages, decreased greatly. Previous study indicated that prostaglandins could also originate from senescent pre-osteoclast and that co-treatment of selective COX-2 inhibitor celecoxib could delay OA progression (Su et al., 2022). In our study, we also found metformin could reduce senescence as well as prostaglandins production induced by high-fat diet. These results highlighted the potential of metformin as a disease-modifying drug that could benefit in different aspects of metabolic syndrome. Interestingly, the inhibition of prostaglandins is also associated with decreased number of osteoblasts and type H vessels, which resulted in improved subchondral bone and cartilage phenotype. These results highlighted the potential of metformin in osteoarthritis treatment.

Pain is one of the main symptoms of OA and was often regarded as an important protective signal that warned the body of existing acute tissue injury and inflammation (Groenewald et al., 2022). OA pain was defined as a dull, chronic, and intermittent pain that could significantly limit daily activity and even harm mental health of the patients (Song et al., 2018). Several important pathways such as cGRP, NGF, TrkA, CCL2, and Wnt/β-catenin signaling pathway were all proved to mediate OA pain (Yu et al., 2022). Metformin was long proved to inhibit pain in metabolic syndrome due to its function in inhibiting inflammation (Baeza-Flores et al., 2020; Na et al., 2021). In this study, we also found that metformin is an effective drug to relieve pain in metabolic syndrome related osteoarthritis. Pain response is one of the most common reasons osteoarthritis patients seek medical care and can significantly affect the quality of life of the patients. Previous reports have shown metformin could inhibit pain transduction by reducing TRPA1 activity in DRG neurons and also TRPA1-mediated calcium influx (Li et al., 2020). In traumatic DMM osteoarthritis model, metformin was found to upregulate phosphorylated and total AMPKα1 expression in DRG tissues and inhibited pain sensitivity (Li et al., 2020). In our study, we for the first time demonstrated the chondroprotective effect of metformin on high-fat diet induced metabolic syndrome related osteoarthritis. Metformin was also able to encourage daily activity and relieve pain response in mice with high-fat diet induced osteoarthritis.

In clinical study, metformin was demonstrated to benefit long-term knee joint outcomes in patients with knee OA and obesity (Wang Y. et al., 2019). COX-2-PGE2 axis is one of the key mechanisms mediating joint destruction and pain response. Recent studies indicated COX-2 inhibitors such as celecoxib could be used to relief pain associated with arthritis and modify disease progression (Su et al., 2022). However, long-term use of COX inhibitor is accompanied by increased risk of GI and cardiovascular events, which may not benefit the overall wellbeing of the patients in a long run (Marsico et al., 2017; Stiller and Hjemdahl, 2022). Moreover, in middle-aged or old patients with osteoarthritis, comorbidities often exist, and different diseases could affect each other, forming a vicious circle. Considering human body as a whole. In such circumstances, a drug like metformin, that is, able to improve cellular metabolism and limit inflammatory response in different organs may appear as a better solution for these patients (Campisi et al., 2019). Randomized controlled clinical trials are needed to determine whether metformin could be used as a potential disease-modifying drug for metabolic syndrome related OA.

## Methods

### Animals and treatment

We purchased the 3-month-old male *C57/BL6* wild-type mice strain from the Model Animal Research Center of Southern Medical University (Guangzhou, China). The general condition of the mice was carefully evaluated and monitored by veterinary examination. All animals were maintained in the animal facility of the Nanfang Hospital, Southern Medical University. The experimental protocol was reviewed and approved by the Nanfang Hospital Animal Ethic Committee (NFYY-2021-1037).

For high-fat diet treatment, mice were placed on a high-fat diet (HFD, 21% fat, Harlan Laboratories, United States) or a regular chow diet (CFD) for designated times. For metformin treatment, metformin (Sigma, 50 mg/kg/d) was diluted in 10% PEG400 and administrated by 100 μl oral gavage daily. Control mice were fed with vehicle.

### Bone sectioning, immunostaining, and histological evaluation

For staining of frozen sections, mice were humanely sacrificed, and knee joints were harvested. After decalcification with 0.5 M EDTA for 2 weeks, bones were immersed in 30% sucrose and 2% polyvinylpyrrolidone (PVP) solution and dehydrate for at least 24 h. The bones were embedded in OCT, and sections were collected for staining. Forty-μm-thick coronal sections were used for immunofluorescence staining. For immunofluorescence staining, we incubated the sections with primary antibodies to COX-2 (abcam, ab179800, 1:200), PGE2 (abcam, ab2318, 1:200), F4/80 (Bio-rad, MCA497RT, 1:200), iNOS (Invitrogen, 14-5920-82, 1:200), CD206 (Bio-rad, MCA2235, 1:200), OSX (abcam, ab22552, 1:300), OCN (Takara, M188, 1:200), EMCN (Santa Cruz, sc-65495, 1:100), CD31 (abcam, FAB3628G, 1:50), overnight at 4°C, and then incubated with second antibodies as described previously (Liu et al., 2021). Nuclei were counterstained with DAPI and observed under an Olympus BX51 microscope.

For Safranin-O& fast green staining, after fixation and decalcification as described previously, the samples were embedded in paraffin, sectioned at 4 μm, followed by Safranin-O& fast green staining. ImageJ software (NIH, United States) was used for quantitative analysis. OARSI scores of the joints’ SOFG staining was performed as described previously (Su et al., 2022).

### qRT-PCR

Total RNA for qRT-PCR was harvested from the mice tibial plateau using RNeasy Mini Kit (QIAGEN) according to the manufacturer’s protocol as described previously (Liu et al., 2023). cDNA was prepared with random primers using the SuperScript First-Strand Synthesis System (Invitrogen, United States) and analyzed with SYBR GreenMaster Mix (QIAGEN) in the thermal cycler. Target-gene expression was normalized to glyceraldehyde 3-phosphate dehydrogenase (GAPDH) messenger RNA, and relative gene expression was assessed using the 2^−ΔΔCT^ method. Primers used for qRT-PCR were as follows: *Cdkn2a* (5′- GAA​AGA​GTT​CGG​GGC​GTT​G-3′) and (5′-GAG​AGC​CAT​CTG​GAG​CAG​CAT-3′); *Cdkn1a* (5′-AGA​AGG​TAC​TTA​CGG​TGT​GGT-3′) and (5′-GAG​AGA​TTT​CCC​GAA​TTG​CAG​T-3′).

### Micro-CT analysis

For all micro-CT analysis, mice knee joints were fixed overnight in 10% formalin at 4°C, and then scanned at a voltage of 55 kVp, a current of 181 μA, and a resolution of 9.0 μm per pixel by high-resolution micro-CT (Bruker MicroCT, Skyscan 1175) (Hu et al., 2020). We used NRecon image reconstruction software, version 1.6 (Bruker MicroCT), CTAn data-analysis software, version 1.9 (Bruker MicroCT) to reconstruct and analyze the parameters of the tibia subchondral bone. 10 coronal images of the medial suchondral bone comartment were selected and used for 3D reconstruction using CTVol v2.0 (Bruker MicroCT).

### Measurement of prostaglandin levels

The concentration of PGE2(R&D systems, KGE004B), PGD2(Cayman chemical, 512011), PGJ2(Novus biologicals, NBP2-64614) and TXA2 (LSBio, LS-F28644) were measured by ELISA assay kits according to manufacter’s instructions. For sample preparation, mice in different treatment groups were sacrificed and tibia subchondral bones were removed carefuly by forceps. The bones were immediately flash-freezed and crushed in liquid nitrogen. Then RIPA buffer was added to the samples and the samples were transferred to a 1.5 ml eppendorff tube. The samples were digested in the tube on a rotator in cold room (4°C) for 30 min and then centrifuged at 12000 RPM at 4°C for 30 min. The supernatant was collected for immediate analysis or stored at −80°C.

### Behavioral tests

Wheel-running activity was recorded by running wheels designed for mice cages (model BIO-ACTIVW-M, Bioseb) as described previously (Ni et al., 2019). Individual mice was placed in the cage with *ad libitum* access to food and water and could run freely. An analyzer was connected to the wheel that could spun both directions and was able to record the mice activity in a cage similar to the mice’s home cage. Parameters including distance traveled, mean speed, and active time for 2 days were recorded for each mouse.

The hind paw withdrawal frequency responding to stimulus was determined by von Frey hairs of 0.7 mN and 3.9 mN (Stoelting, Wood Dale, IL). Mice were pre-conditioned for the environment for 30 min before testing. Von Frey hairs were applied to the mid-plantar surface of the mice hind paw which was in contact with the floor for ten times. The withdrawal frequency of the mice hind paw was recorded and calculated as the percentage of withdrawal times in response to ten von frey hair applications.

## Statistics

Data are presented as means ± standard errors of the mean. For multiple comparisons, one-way analysis of variance (ANOVA) with Bonferroni *post hoc* test was used. All data were normally distributed and had similar variation between groups. Statistical analysis was performed using SAS, version 9.3, software (SAS Institute, NC). *P* < 0.05 was deemed significant.

## Data Availability

The data that support the findings of this study are available within the article or from the corresponding author upon reasonable request.

## References

[B1] Allen K. D. Thoma L. M. Golightly Y. M. (2022). Epidemiology of osteoarthritis. Osteoarthr. Cartil. 30 (2), 184–195. 10.1016/j.joca.2021.04.020 PMC10735233 34534661

[B2] Baeza-Flores G. Guzman-Priego C. G. Parra-Flores L. I. Murbartian J. Torres-Lopez J. E. Granados-Soto V. (2020). Metformin: A prospective alternative for the treatment of chronic pain. Front. Pharmacol. 11, 558474. 10.3389/fphar.2020.558474 33178015 PMC7538784

[B3] Batushansky A. Zhu S. Komaravolu R. K. South S. Mehta-D'Souza P. Griffin T. M. (2022). Fundamentals of oa. An initiative of osteoarthritis and cartilage. Obesity and metabolic factors in oa. Osteoarthr. Cartil. 30 (4), 501–515. 10.1016/j.joca.2021.06.013 PMC8926936 34537381

[B4] Block J. A. (2014). Osteoarthritis: Oa guidelines: Improving care or merely codifying practice? Nat. Rev. Rheumatol. 10 (6), 324–326. 10.1038/nrrheum.2014.61 24752185

[B5] Brunner A. M. Henn C. M. Drewniak E. I. Lesieur-Brooks A. Machan J. Crisco J. J. (2012). High dietary fat and the development of osteoarthritis in a rabbit model. Osteoarthr. Cartil. 20 (6), 584–592. 10.1016/j.joca.2012.02.007 22353745

[B6] Campisi J. Kapahi P. Lithgow G. J. Melov S. Newman J. C. Verdin E. (2019). From discoveries in ageing research to therapeutics for healthy ageing. Nature 571 (7764), 183–192. 10.1038/s41586-019-1365-2 31292558 PMC7205183

[B7] Chen H. Hu B. Lv X. Zhu S. Zhen G. Wan M. (2019). Prostaglandin e2 mediates sensory nerve regulation of bone homeostasis. Nat. Commun. 10 (1), 181. 10.1038/s41467-018-08097-7 30643142 PMC6331599

[B8] Collins K. H. Herzog W. MacDonald G. Z. Reimer R. A. Rios J. L. Smith I. C. (2018). Obesity, metabolic syndrome, and musculoskeletal disease: Common inflammatory pathways suggest a central role for loss of muscle integrity. Front. Physiol. 9, 112. 10.3389/fphys.2018.00112 29527173 PMC5829464

[B9] Conaghan P. G. Cook A. D. Hamilton J. A. Tak P. P. (2019). Therapeutic options for targeting inflammatory osteoarthritis pain. Nat. Rev. Rheumatol. 15 (6), 355–363. 10.1038/s41584-019-0221-y 31068673

[B10] Courties A. Sellam J. Berenbaum F. (2017). Metabolic syndrome-associated osteoarthritis. Curr. Opin. Rheumatol. 29 (2), 214–222. 10.1097/BOR.0000000000000373 28072592

[B11] Cui Z. Crane J. Xie H. Jin X. Zhen G. Li C. (2016). Halofuginone attenuates osteoarthritis by inhibition of tgf-beta activity and h-type vessel formation in subchondral bone. Ann. Rheum. Dis. 75 (9), 1714–1721. 10.1136/annrheumdis-2015-207923 26470720 PMC5013081

[B12] Furuta K. Tang X. Islam S. Tapia A. Chen Z. B. Ibrahim S. H. (2023). Endotheliopathy in the metabolic syndrome: Mechanisms and clinical implications. Pharmacol. Ther. 244, 108372. 10.1016/j.pharmthera.2023.108372 36894027 PMC10084912

[B13] Groenewald C. B. Murray C. B. Battaglia M. Scaini S. Quinn P. D. (2022). Prevalence of pain management techniques among adults with chronic pain in the United States, 2019. JAMA Netw. Open 5 (2), e2146697. 10.1001/jamanetworkopen.2021.46697 35129599 PMC8822381

[B14] Gross J. B. Guillaume C. Gegout-Pottie P. Mainard D. Presle N. (2014). Synovial fluid levels of adipokines in osteoarthritis: Association with local factors of inflammation and cartilage maintenance. Biomed. Mater Eng. 24 (1), 17–25. 10.3233/BME-140970 24928914

[B15] Hattori Y. Suzuki K. Hattori S. Kasai K. (2006). Metformin inhibits cytokine-induced nuclear factor kappab activation via amp-activated protein kinase activation in vascular endothelial cells. Hypertension 47 (6), 1183–1188. 10.1161/01.HYP.0000221429.94591.72 16636195

[B16] Hayes M. E. Rai A. Cooper R. G. Bayley D. Freemont A. J. Mawer E. B. (1992). Inhibition by prostaglandin e1 and e2 of 1,25-dihydroxyvitamin d3 synthesis by synovial fluid macrophages from arthritic joints. Ann. Rheum. Dis. 51 (5), 632–637. 10.1136/ard.51.5.632 1616328 PMC1005696

[B17] Hu B. Lv X. Chen H. Xue P. Gao B. Wang X. (2020). Sensory nerves regulate mesenchymal stromal cell lineage commitment by tuning sympathetic tones. J. Clin. Invest. 130 (7), 3483–3498. 10.1172/JCI131554 32191640 PMC7324175

[B18] Hunter D. J. Bierma-Zeinstra S. (2019). Osteoarthritis. Lancet 393 (10182), 1745–1759. 10.1016/S0140-6736(19)30417-9 31034380

[B19] Isoda K. Young J. L. Zirlik A. MacFarlane L. A. Tsuboi N. Gerdes N. (2006). Metformin inhibits proinflammatory responses and nuclear factor-kappab in human vascular wall cells. Arterioscler. Thromb. Vasc. Biol. 26 (3), 611–617. 10.1161/01.ATV.0000201938.78044.75 16385087

[B20] Jeon O. H. Kim C. Laberge R. Demaria M. Rathod S. Vasserot A. P. (2017). Local clearance of senescent cells attenuates the development of post-traumatic osteoarthritis and creates a pro-regenerative environment. Nat. Med. 23 (6), 775–781. 10.1038/nm.4324 28436958 PMC5785239

[B21] Kassi E. Pervanidou P. Kaltsas G. Chrousos G. (2011). Metabolic syndrome: Definitions and controversies. BMC Med. 9, 48. 10.1186/1741-7015-9-48 21542944 PMC3115896

[B22] Kulkarni A. S. Gubbi S. Barzilai N. (2020). Benefits of metformin in attenuating the hallmarks of aging. Cell Metab. 32 (1), 15–30. 10.1016/j.cmet.2020.04.001 32333835 PMC7347426

[B23] Li D. Ruan G. Zhang Y. Zhao Y. Zhu Z. Ou Q. (2022). Metformin attenuates osteoarthritis by targeting chondrocytes, synovial macrophages and adipocytes. Rheumatol. Oxf. 62, 1652–1661. 10.1093/rheumatology/keac467 35984286

[B24] Li J. Zhang B. Liu W. X. Lu K. Pan H. Wang T. (2020). Metformin limits osteoarthritis development and progression through activation of ampk signalling. Ann. Rheum. Dis. 79 (5), 635–645. 10.1136/annrheumdis-2019-216713 32156705 PMC7213329

[B25] Li Z. Liu L. Yang Y. Zheng H. Cai Y. Ma Y. (2022). Metformin ameliorates senescence of adipose-derived mesenchymal stem cells and attenuates osteoarthritis progression via the ampk-dependent autophagy pathway. Oxid. Med. Cell. Longev. 2022, 4620254. 10.1155/2022/4620254 35693701 PMC9187432

[B26] Liedert A. Wagner L. Seefried L. Ebert R. Jakob F. Ignatius A. (2010). Estrogen receptor and wnt signaling interact to regulate early gene expression in response to mechanical strain in osteoblastic cells. Biochem. Biophys. Res. Commun. 394 (3), 755–759. 10.1016/j.bbrc.2010.03.065 20227388

[B27] Liu S. Y. Zhu W. T. Chen B. W. Chen Y. H. Ni G. X. (2020). Bidirectional association between metabolic syndrome and osteoarthritis: A meta-analysis of observational studies. Diabetol. Metab. Syndr. 12, 38. 10.1186/s13098-020-00547-x 32399062 PMC7204053

[B28] Liu X. Chai Y. Liu G. Su W. Guo Q. Lv X. (2021). Osteoclasts protect bone blood vessels against senescence through the angiogenin/plexin-b2 axis. Nat. Commun. 12 (1), 1832. 10.1038/s41467-021-22131-1 33758201 PMC7987975

[B29] Liu X. Gu Y. Kumar S. Amin S. Guo Q. Wang J. (2023). Oxylipin-PPARγ-initiated adipocyte senescence propagates secondary senescence in the bone marrow. Cell Metab. 35 (4), 667–684.e6. 10.1016/j.cmet.2023.03.005 37019080 PMC10127143

[B30] Lv X. Gao F. Cao X. (2022). Skeletal interoception in bone homeostasis and pain. Cell Metab. 34 (12), 1914–1931. 10.1016/j.cmet.2022.09.025 36257317 PMC9742337

[B31] Marsico F. Paolillo S. Filardi P. P. (2017). Nsaids and cardiovascular risk. J. Cardiovasc Med. Hagerst. 18 Suppl 1: Special Issue on The State of the Art for the Practicing Cardiologist: The 2016 Conoscere E Curare Il Cuore (CCC) Proceedings from the CLI Foundation, e40–e43. 10.2459/JCM.0000000000000443 27652819

[B32] Martel-Pelletier J. Barr A. J. Cicuttini F. M. Conaghan P. G. Cooper C. Goldring M. B. (2016). Osteoarthritis. Nat. Rev. Dis. Prim. 2, 16072. 10.1038/nrdp.2016.72 27734845

[B33] Minamizaki T. Yoshiko Y. Kozai K. Aubin J. E. Maeda N. (2009). Ep2 and ep4 receptors differentially mediate mapk pathways underlying anabolic actions of prostaglandin e2 on bone formation in rat calvaria cell cultures. Bone 44 (6), 1177–1185. 10.1016/j.bone.2009.02.010 19233324

[B34] Misra D. Fielding R. A. Felson D. T. Niu J. Brown C. Nevitt M. (2019). Risk of knee osteoarthritis with obesity, sarcopenic obesity, and sarcopenia. Arthritis Rheumatol. 71 (2), 232–237. 10.1002/art.40692 30106249 PMC6374038

[B35] Mohajer B. Kwee R. M. Guermazi A. Berenbaum F. Wan M. Zhen G. (2021). Metabolic syndrome and osteoarthritis distribution in the hand joints: A propensity score matching analysis from the osteoarthritis initiative. J. Rheumatol. 48 (10), 1608–1615. 10.3899/jrheum.210189 34329188

[B36] Mohammed I. Hollenberg M. D. Ding H. Triggle C. R. (2021). A critical review of the evidence that metformin is a putative anti-aging drug that enhances healthspan and extends lifespan. Front. Endocrinol. (Lausanne) 12, 718942. 10.3389/fendo.2021.718942 34421827 PMC8374068

[B37] Montalvany-Antonucci C. C. Zicker M. C. Ferreira A. Macari S. Ramos-Junior E. S. Gomez R. S. (2018). High-fat diet disrupts bone remodeling by inducing local and systemic alterations. J. Nutr. Biochem. 59, 93–103. 10.1016/j.jnutbio.2018.06.006 29986312

[B38] Na H. S. Kwon J. Y. Lee S. Y. Lee S. H. Lee A. R. Woo J. S. (2021). Metformin attenuates monosodium-iodoacetate-induced osteoarthritis via regulation of pain mediators and the autophagy-lysosomal pathway. Cells 10 (3), 681. 10.3390/cells10030681 33808727 PMC8003384

[B39] Ni S. Ling Z. Wang X. Cao Y. Wu T. Deng R. (2019). Sensory innervation in porous endplates by netrin-1 from osteoclasts mediates pge2-induced spinal hypersensitivity in mice. Nat. Commun. 10 (1), 5643. 10.1038/s41467-019-13476-9 31822662 PMC6904550

[B40] Puenpatom R. A. Victor T. W. (2009). Increased prevalence of metabolic syndrome in individuals with osteoarthritis: An analysis of nhanes iii data. Postgrad. Med. 121 (6), 9–20. 10.3810/pgm.2009.11.2073 19940413

[B41] Qiao J. Wu Y. Ren Y. (2021). The impact of a high fat diet on bones: Potential mechanisms. Food Funct. 12 (3), 963–975. 10.1039/d0fo02664f 33443523

[B42] Raisz L. G. (1999). Prostaglandins and bone: Physiology and pathophysiology. Osteoarthr. Cartil. 7 (4), 419–421. 10.1053/joca.1998.0230 10419786

[B43] Sanchez-Santos M. T. Judge A. Gulati M. Spector T. D. Hart D. J. Newton J. L. (2019). Association of metabolic syndrome with knee and hand osteoarthritis: A community-based study of women. Semin. Arthritis Rheum. 48 (5), 791–798. 10.1016/j.semarthrit.2018.07.007 30172470

[B44] Sansone V. Applefield R. C. De Luca P. Pecoraro V. Gianola S. Pascale W. (2019). Does a high-fat diet affect the development and progression of osteoarthritis in mice? A systematic review. Bone Jt. Res. 8 (12), 582–592. 10.1302/2046-3758.812.BJR-2019-0038.R1 PMC6946912 31934329

[B45] Schadler P. Lohberger B. Stundl N. Stradner M. H. Glanzer D. Sadoghi P. (2021). The effect of body mass index and metformin on matrix gene expression in arthritic primary human chondrocytes. Cartilage 13 (2), 1004S–1018S. 10.1177/1947603520962558 33025801 PMC8804722

[B46] Song J. Chang A. H. Chang R. W. Lee J. Pinto D. Hawker G. (2018). Relationship of knee pain to time in moderate and light physical activities: Data from osteoarthritis initiative. Semin. Arthritis Rheum. 47 (5), 683–688. 10.1016/j.semarthrit.2017.10.005 29103557 PMC5866183

[B47] Stiller C. O. Hjemdahl P. (2022). Lessons from 20 years with cox-2 inhibitors: Importance of dose-response considerations and fair play in comparative trials. J. Intern. Med. 292 (4), 557–574. 10.1111/joim.13505 35585779

[B48] Su W. Liu G. Liu X. Zhou Y. Sun Q. Zhen G. (2020). Angiogenesis stimulated by elevated pdgf-bb in subchondral bone contributes to osteoarthritis development. JCI Insight 5, e135446. 10.1172/jci.insight.135446 32208385 PMC7205438

[B49] Su W. Liu G. Mohajer B. Wang J. Shen A. Zhang W. (2022). Senescent preosteoclast secretome promotes metabolic syndrome associated osteoarthritis through cyclooxygenase 2. eLife 11, e79773. 10.7554/eLife.79773 35881544 PMC9365389

[B50] Sun Q. Zhen G. Li T. P. Guo Q. Li Y. Su W. (2021). Parathyroid hormone attenuates osteoarthritis pain by remodeling subchondral bone in mice. eLife 10, e66532. 10.7554/eLife.66532 33646122 PMC8012060

[B51] Terkeltaub R. Yang B. Lotz M. Liu-Bryan R. (2011). Chondrocyte AMP-activated protein kinase activity suppresses matrix degradation responses to proinflammatory cytokines interleukin-1β and tumor necrosis factor α. Arthritis Rheum. 63 (7), 1928–1937. 10.1002/art.30333 21400477 PMC3128233

[B52] Tu M. Yang M. Yu N. Zhen G. Wan M. Liu W. (2019). Inhibition of cyclooxygenase-2 activity in subchondral bone modifies a subtype of osteoarthritis. Bone Res. 7, 29. 10.1038/s41413-019-0071-x 31666999 PMC6804921

[B53] Wang C. Yang Y. Zhang Y. Liu J. Yao Z. Zhang C. (2019). Protective effects of metformin against osteoarthritis through upregulation of sirt3-mediated pink1/parkin-dependent mitophagy in primary chondrocytes. Biosci. Trends. 12 (6), 605–612. 10.5582/bst.2018.01263 30584213

[B54] Wang Y. Hussain S. M. Wluka A. E. Lim Y. Z. Abram F. Pelletier J. P. (2019). Association between metformin use and disease progression in obese people with knee osteoarthritis: Data from the osteoarthritis initiative-a prospective cohort study. Arthritis Res. Ther. 21 (1), 127. 10.1186/s13075-019-1915-x 31126352 PMC6534888

[B55] Xie C. Chen Q. (2019). Adipokines: New therapeutic target for osteoarthritis? Curr. Rheumatol. Rep. 21 (12), 71. 10.1007/s11926-019-0868-z 31813080 PMC7291783

[B56] Xing H. Liang C. Wang C. Xu X. Hu Y. Qiu B. (2022). Metformin mitigates cholesterol accumulation via the ampk/sirt1 pathway to protect osteoarthritis chondrocytes. Biochem. Biophys. Res. Commun. 632, 113–121. 10.1016/j.bbrc.2022.09.074 36206595

[B57] Yu H. Huang T. Lu W. W. Tong L. Chen D. (2022). Osteoarthritis pain. Int. J. Mol. Sci. 23 (9), 4642. 10.3390/ijms23094642 35563035 PMC9105801

[B58] Zhen G. Wen C. Jia X. Li Y. Crane J. L. Mears S. C. (2013). Inhibition of tgf-beta signaling in mesenchymal stem cells of subchondral bone attenuates osteoarthritis. Nat. Med. 19 (6), 704–712. 10.1038/nm.3143 23685840 PMC3676689

[B59] Zhu S. Zhu J. Zhen G. Hu Y. An S. Li Y. (2019). Subchondral bone osteoclasts induce sensory innervation and osteoarthritis pain. J. Clin. Invest. 129 (3), 1076–1093. 10.1172/JCI121561 30530994 PMC6391093

